# Beverage Consumption Patterns, Beverage-Derived Energy Intake, and Adherence to Fluid Intake Recommendations Among Polish Adolescents: A Cross-Sectional Study Using the BEVQ-PL Questionnaire

**DOI:** 10.3390/nu18172784

**Published:** 2026-08-26

**Authors:** Marek Zborowski, Magdalena Skotnicka

**Affiliations:** 1 The Faculty of Medicine and Health Sciences, University of Applied Sciences in Nowy Sącz, Kościuszki 2G, 33-300 Nowy Sącz, Poland; mzborowski@ans-ns.edu.pl; 2 Department of Commodity Science, Faculty of Health Sciences, Medical University of Gdansk, 7 Debinki Street, 80-210 Gdansk, Poland

**Keywords:** adolescents, beverage consumption, hydration recommendations, adequate intake, BEVQ-PL

## Abstract

**Background/Objectives:** Different organizations have established fluid intake recommendations for adolescents; however, studies assessing adherence typically rely on a single reference system, limiting comparisons across populations. This study assessed beverage consumption patterns and beverage-derived energy intake among Polish adolescents and examined how adherence estimates differed when recommendations issued by the European Food Safety Authority (EFSA), the Institute of Medicine (IOM), and the Polish National Institute of Public Health—National Institute of Hygiene (NIH) were applied. **Methods:** A cross-sectional study was conducted among 1644 secondary school students aged 14–18 years. Beverage consumption (mL/day) and beverage-derived energy intake (kcal/day) were assessed using the validated Polish Beverage Intake Questionnaire (BEVQ-PL). Adherence was defined as achieving at least 80% of the age- and sex-specific Adequate Intake (AI) from beverages, assuming that beverages provide approximately 80% of total daily water intake. **Results:** Water had the highest median daily intake (960 mL/day). Males consumed significantly more water than females (1080 vs. 720 mL/day; *p* = 0.037), whereas females consumed significantly more reduced-fat milk (2%), skim milk, plant-based and flavored milk beverages, 100% vegetable juice, and coffee with milk and sugar. Adherence differed according to the recommendation system, reaching 65.2% (EFSA), 50.6% (IOM), and 65.8% (NIH). Female sex was independently associated with higher adherence across all three reference systems, whereas older age was associated with lower adherence only according to the NIH criteria and overweight/obesity only according to the IOM criteria. **Conclusions:** Estimated adherence to fluid intake recommendations varied substantially according to the reference system applied. These findings highlight the importance of explicitly reporting the selected reference criteria when assessing and comparing the adequacy of beverage-derived fluid intake among adolescents.

## 1. Introduction

Water is essential for maintaining physiological and cognitive functions and supports numerous processes involved in human homeostasis [1,2,3]. Even mild dehydration may adversely affect thermoregulation and physical performance [4]. Adequate water intake is particularly important during childhood and adolescence because physiological water requirements vary with age, sex, physical activity, and environmental conditions [5,6,7,8,9]. Beyond the quantity of fluids consumed, beverage type is an important component of dietary behavior and may influence health outcomes [10]. In particular, the widespread consumption of sugar-sweetened beverages (SSBs) among young people remains a public health concern because of their potential contribution to excessive energy intake and obesity risk [11,12]. Current research has focused disproportionately on the impact of diet on health outcomes, while often overlooking hydration as a critical determinant of health. Furthermore, sustainable lifestyle modifications—particularly those related to dietary habits and hydration practices—remain challenging to implement and monitor over time [13]. There is no universally accepted standard for recommended water intake in children and adolescents. In both clinical practice and research, the most commonly applied reference values are the Adequate Intake (AI) recommendations established by the European Food Safety Authority (EFSA), the Institute of Medicine (IOM), and the Polish National Institute of Public Health—National Institute of Hygiene (NIH). Although all three organizations define AI as the level of water intake considered sufficient to maintain adequate hydration and normal physiological function in healthy populations, the recommended intake values differ across these reference systems [2].

The age- and sex-specific AI values differ between the EFSA, IOM, and NIH reference systems. A direct comparison of the reference values applicable to adolescents in the age range covered by the present study is presented in Table 1 [14,15,16].

Assessing adherence according to multiple reference systems is important because differences in their age- and sex-specific AI values may result in different estimates of the proportion of adolescents classified as meeting fluid intake recommendations, thereby affecting comparisons between studies and populations [2]. It should be emphasized that all of the above recommendations refer to Total Water Intake (TWI), which includes water obtained from both beverages and foods. However, in European populations, approximately 80% of total water intake is derived from beverages, with the remaining proportion provided by foods [17]. Consequently, epidemiological studies assessing beverage consumption alone commonly use the achievement of at least 80% of the AI as an indicator of adequate fluid intake from beverages. This approach has been widely adopted in previous epidemiological research [18].

Despite the growing body of research on beverage consumption among children and adolescents, several important knowledge gaps remain. Previous studies have primarily focused on overall beverage intake or selected beverage categories, while relatively few have simultaneously evaluated beverage consumption patterns, energy intake from beverages, and estimated adherence to reference intake values based on beverage intake [2,19].

Furthermore, most studies have assessed adherence using a single set of reference values, whereas studies reporting adherence according to multiple reference systems, including EFSA, IOM, and NIH, remain limited.

In addition, relatively little is known about the demographic and anthropometric factors associated with adherence to these recommendations among adolescents. Simultaneous application of all three reference systems enables adherence estimates to be reported separately according to each set of reference values. Such evidence may improve the interpretation of epidemiological findings and support the development of public health strategies and nutrition education programs aimed at promoting adequate fluid intake among adolescents [2].

The validated Polish version of the Beverage Intake Questionnaire (BEVQ-PL) provides a standardized approach to assessing beverage consumption among Polish adolescents [19]. Its application in the present study enabled the simultaneous assessment of individual beverage categories and beverage-derived energy intake within the same adolescent population. The scientific contribution of the present study lies in its integrated evaluation of beverage consumption patterns, beverage-derived energy intake, and adherence to fluid intake recommendations within the same adolescent population, together with a direct comparison of three reference systems (EFSA, IOM, and NIH). Applying all three systems to the same population allows the extent to which the choice of reference criteria influences estimates of adherence to be evaluated directly. In addition, the study examines whether demographic, anthropometric, and lifestyle characteristics are independently associated with adherence across the different reference systems. The aim of this study was therefore to assess beverage consumption patterns and energy intake from beverages among Polish adolescents using the validated BEVQ-PL, to compare adherence to fluid intake recommendations across the EFSA, IOM, and NIH reference systems, and to identify factors independently associated with adherence to these recommendations. Based on the existing literature and the identified research gaps, the following hypotheses were formulated:

**H1.**  *Sex is independently associated with adherence to fluid intake recommendations.*

**H2.**  *Age and body mass index (BMI) are associated with the likelihood of meeting these recommendations.*

## 2. Materials and Methods

### 2.1. Study Design

This study was cross-sectional in nature and was conducted among secondary school students in Poland aged 14–18. The manuscript was prepared in accordance with the Strengthening the Reporting of Observational Studies in Epidemiology (STROBE) guidelines for reporting cross-sectional observational studies [20].

The study was conducted from October 2025 to June 2026 in secondary schools in the Małopolska Voivodeship. Data were collected successively throughout the school year; however, the timing of data collection was not incorporated into the statistical analyses, and potential seasonal variation in beverage consumption cannot therefore be excluded. Poland lies within the temperate climate zone; therefore, although regional differences in weather conditions occur, Małopolska does not represent a distinct climatic zone compared with other regions of the country.

### 2.2. Study Participants

The study was conducted in secondary schools located in the Małopolska Voivodeship. The region is located in southern Poland and comprises both urban and rural areas. Its adolescent population includes students from diverse residential environments, making the region a suitable setting for examining beverage consumption patterns among secondary school students. The Małopolska Voivodeship was selected as the study area due to the feasibility of conducting a large-scale school-based study across multiple secondary schools in the region. Schools were recruited through direct contact between the author and the school principals. Schools were selected using a non-probability convenience sampling approach based on their accessibility to the researchers. A total of 16 schools were invited to participate, of which 15 agreed and provided complete data. After obtaining the principal’s consent, all students meeting the inclusion criteria were invited to participate in the study. Before the study began, participants, and in the case of minors, their parents or legal guardians, received detailed information regarding the purpose and procedure of the study and provided written informed consent to participate. Sample size was estimated according to recommendations for cross-sectional studies, using the method described by Cochran [21]. A confidence level of 95%, a maximum error of estimate of 5%, and a conservative assumption of a 50% prevalence of the analyzed characteristic in the population were adopted as the reference population. In total, 2,083,060 Polish residents aged 14–18, according to data from the Central Statistical Office for 2025, were assumed [22]. The minimum required sample size was 384 individuals. The final sample size significantly exceeded the minimum required size, which increased the precision of the obtained estimates and the statistical power of the conducted analyses.

The following inclusion criteria were used in the study: age 14–18 years, secondary school student status, informed consent, and completion of the BEVQ-PL questionnaire. Individuals who did not provide information about sex (*n* = 85) were excluded from the analysis, as this prevented the assignment of appropriate fluid intake reference values in accordance with the recommendations of EFSA, IOM, and NIH. Participants with biologically implausible anthropometric values or internally inconsistent data (e.g., body weight, height, or BMI outside the realistic range) that could indicate data entry errors (*n* = 15) were also excluded. Overall, 131 individuals did not meet the eligibility criteria or were excluded from the analysis. Ultimately, 1644 participants were included in the statistical analyses.

Place of residence and physical activity were assessed using self-reported questions. Place of residence was categorized as a rural area or urban; urban areas were further classified according to population size as <50,000, 50,000–150,000, or 150,000–500,000 inhabitants. Physical activity was assessed based on the self-reported frequency of engaging in physical activity and categorized as no physical activity, occasionally (≤1 time/month), at least once per week, or several times per week. The study population did not include adolescents engaged in competitive sports training; therefore, the physical activity categories reflected habitual non-competitive physical activity.

The BEVQ-PL questionnaire was completed during school hours in the presence of trained research team members, who provided consistent instructions on how to complete the survey and answered participants’ technical questions without influencing the content of their responses. This procedure was intended to reduce the number of missing data and ensure the most uniform conditions for conducting the study. The surveys were anonymous, intended to reduce the impact of social pressure and increase the reliability of responses [23].

### 2.3. Assessment of Anthropometric Parameters

To reduce measurement errors, all anthropometric assessments were performed by trained research team members according to standardized procedures and current anthropometric standards. Body height was determined to the nearest 0.1 cm using a stadiometer (Seca 213, Hamburg, Germany). Participants stood barefoot on a platform, leaning against the stadiometer’s pole, looking straight ahead, and keeping their upper limbs alongside their bodies. Body weight was determined to the nearest 0.1 kg using an InBody 120 device (InBody Co., Ltd., Seoul, Republic of Korea). All measurements were taken before noon, with participants barefoot, wearing light clothing, and after emptying their pockets of items that could influence the measurement results.

Due to the age of the study population, nutritional status was classified using the Polish OLA and OLAF percentile charts, developed for children and adolescents [24]. The OLA and OLAF reference charts were selected because they were developed specifically for the Polish pediatric population and therefore provide population-specific reference values for BMI according to age and sex. For each participant, BMI was compared to the appropriate percentile values for sex and age. Based on this, participants were classified into two categories: underweight or normal weight (<85th percentile) and overweight (≥85th percentile), which includes overweight and obesity. This two-category division was adopted to ensure adequate group sizes and increase the stability of statistical analyses.

### 2.4. Assessment of Beverage Consumption

The self-administered Polish version of the Beverage Intake Questionnaire (BEVQ-PL) [19], an adapted and validated version of the original BEVQ-15 [25,26], was used to assess habitual beverage consumption. The questionnaire simultaneously assesses the frequency of consumption and serving size of individual beverage groups, allowing for the estimation of average daily intake and energy intake from beverages. The BEVQ-PL included the following beverage groups: water, 100% fruit juice, sweetened fruit drinks and lemonades, 100% vegetable juice, whole milk (3.2% fat), reduced-fat milk (2%), skim milk, plant-based and flavored milk beverages, sugar-sweetened beverages (SSBs, e.g., soda, cola), diet beverages with sweeteners (e.g., zero-calorie beverages), energy drinks, sweetened tea, black coffee or tea, coffee with milk and sugar, smoothies, and protein drinks.

Alcoholic beverages (beer, spirits, alcoholic mixed drinks, wine) were not included in the assessment of total daily fluid intake or adherence to adequate fluid intake recommendations because current dietary reference values refer exclusively to water and non-alcoholic beverages. Although the questionnaire included a separate item for non-alcoholic beer, this category was not considered in the present analyses to ensure consistency with the evaluated dietary recommendations.

For each beverage category, participants reported their consumption over the past month, including both the frequency of consumption (answering the question “how often”) and the typical volume of a single serving (“how much each time”). To facilitate accurate portion size estimation, the questionnaire included sample volumes corresponding to the most common beverage packaging. The questionnaire assessed habitual beverage consumption, not consumption over a single day, proving that the results reflected participants’ typical dietary behavior.

The Polish version of the BEVQ-PL questionnaire had previously undergone cultural adaptation and preliminary psychometric validation among Polish adolescents. The results demonstrated moderate to good test–retest reliability for most beverage categories and supported the use of the BEVQ-PL for assessing beverage intake at the group level. The validated version of the questionnaire was used in this study.

Prior to statistical analysis, beverage consumption data were checked for completeness, coding errors, and internal consistency. Responses were verified against the predefined frequency and portion-size categories of the BEVQ-PL questionnaire, including consistency between the reported frequency and typical serving size for each beverage category. Daily beverage intake (mL/day) was then calculated according to the BEVQ-PL scoring procedure. The distributions and ranges of the calculated intake values were inspected to identify potentially erroneous or implausible observations. Extreme intake values were not automatically excluded solely on the basis of their magnitude; they were retained when they resulted from valid and internally consistent combinations of the predefined BEVQ-PL response categories.

As an additional sensitivity analysis, selected beverage categories characterized by markedly right-skewed distributions and extreme observations were winsorized at the 99th percentile to assess whether extreme intake values materially affected the observed between-sex differences. The statistical conclusions remained unchanged after winsorization; therefore, the original non-winsorized values were retained in the primary analyses.

### 2.5. Calculating Daily Beverage Consumption and Energy Intake

To quantify beverage consumption, responses regarding beverage consumption frequency were converted to daily equivalents according to the published BEVQ-PL methodology [19]. Average daily consumption of individual beverage categories (mL/day) was calculated based on self-reported consumption frequency and serving size, in accordance with the published BEVQ-PL validation methodology. For each beverage category, daily intake (mL/day) was calculated by multiplying the reported serving volume (mL) by the corresponding daily frequency of consumption. Total daily beverage consumption was calculated by summing intake across all analyzed beverage categories. Daily energy intake (kcal/day) for each beverage category was calculated by multiplying the estimated daily intake (mL/day) by the corresponding energy density coefficient (kcal/mL) presented in Supplementary Table S1.

### 2.6. Assessment of Adherence to Fluid Intake Recommendations

Based on the calculated total daily beverage intake, adherence to the EFSA, IOM, and NIH recommendations was defined as achieving at least 80% of the age- and sex-specific AI. The age- and sex-specific AI reference values used in the analysis are summarized in Table 1. The three reference systems were selected to provide complementary national and international perspectives on fluid intake adequacy. The Polish national recommendations were considered the most directly applicable reference for the study population, whereas the EFSA recommendations provided a European reference framework and the IOM recommendations facilitated comparison with international studies using American reference values.

Because the AI values determined by EFSA, IOM, and NIH refer to total water intake, including both water from beverages and food, the results obtained using the BEVQ-PL questionnaire were not directly compared to 100% of the AI. This questionnaire assesses only beverage consumption, whereas on average, approximately 20% of total water intake comes from solid foods. Consequently, it was assumed that beverage consumption corresponding to at least 80% of the AI value indicates compliance with the fluid intake recommendations [17]. The 80% threshold is therefore not arbitrary but reflects a population-level estimate of the average contribution of beverages to total water intake. However, this proportion was not measured individually in the present study and may vary between participants depending on the water content of their diet. Therefore, the ≥80% AI threshold should be regarded as a pragmatic epidemiological approximation of beverage-based adherence rather than a direct individual-level assessment of compliance with total water AI recommendations. Consequently, some misclassification of individual participants as meeting or not meeting the recommendations cannot be excluded.

For each participant, the degree of compliance was calculated using the following formula:

$$Adequate\;Intake\;\left(AI\right)\;Achievement=\frac{\mathrm{Daily}\;\mathrm{beverage}\;\mathrm{intake}\;(\mathrm{mL})}{AI\;value\;(mL)}\times 100$$

Subsequently, adherence to each set of fluid intake recommendations (EFSA, IOM, and NIH) was coded as a separate dichotomous variable: 0 = intake < 80% of the AI value; 1 = intake ≥ 80% of the AI value.

### 2.7. Statistical Analysis

Statistical analyses were performed using Statistica version 14.1.0.4 (TIBCO Software Inc., San Ramon, CA, USA). The normality of continuous variables was assessed using the Shapiro–Wilk test. Continuous variables are presented as mean ± standard deviation (SD) or median (interquartile range, IQR), as appropriate. Age, body weight, height, and body mass index (BMI) are presented as mean ± SD as standard descriptive characteristics of the study population to facilitate comparison with previous studies. As most continuous variables were not normally distributed, comparisons between two independent groups were performed using the Mann–Whitney U test. As a sensitivity analysis, the intake from each beverage category and beverage-derived energy intake were additionally expressed as a percentage of total daily beverage intake and total beverage-derived energy intake, respectively. Sex differences in these relative contributions were assessed using the Mann–Whitney U test. Similarly, energy intake from each beverage category was expressed as a percentage of total daily energy intake from beverages to assess whether sex differences persisted after accounting for differences in total beverage-derived energy intake. Differences between females and males were assessed using the Mann–Whitney U test.

Categorical variables are presented as frequencies and percentages and were compared using Pearson’s chi-square test. Effect sizes were quantified using Cramér’s V.

Additionally, a subgroup analysis of energy drink consumption frequency was performed. Participants consuming energy drinks 2–3 times per week were distinguished from those reporting consumption once per week or less frequently. Differences according to sex, age group, and BMI category were assessed using Pearson’s chi-square test. A multivariable logistic regression model including sex, age group, and BMI category was additionally used to identify factors independently associated with energy drink consumption at least 2–3 times per week. Multivariable logistic regression was used to examine factors independently associated with adherence to fluid intake recommendations. Three separate models were fitted for adherence according to the EFSA, IOM, and NIH recommendations. Sex, age group, BMI category, and physical activity were selected a priori based on their relevance to fluid requirements and beverage-related behaviors in adolescents. For each model, adherence to the recommended fluid intake served as the dependent variable (0 = did not meet the recommendation; 1 = met the recommendation). Independent variables included sex, age group (14–15 and 16–18 years), BMI category (underweight/normal weight vs. overweight/obesity), and physical activity (no physical activity, occasionally [≤1 time/month], at least once per week, and several times per week). Results are reported as odds ratios (ORs) with 95% confidence intervals (95% CIs).

### 2.8. Ethics Statement

The study was conducted in accordance with the principles of the Declaration of Helsinki and was approved by the Bioethics Committee of the Medical University of Gdańsk (approval No. KB/432/2025 from the day 13 October 2025). Participation was voluntary and anonymous. Written informed consent was obtained from all individuals prior to enrollment. For those younger than 18 years, written informed assent was obtained from the adolescents themselves, together with written informed consent from their parents or legal guardians.

## 3. Results

A total of 1644 adolescents were included in the study, comprising 660 females (40.1%) and 984 males (59.9%). The mean age of the participants was 16.4 ± 1.1 years. Females were slightly older than males (16.4 ± 1.1 vs. 16.3 ± 1.1 years, *p* = 0.003). Males had a significantly higher body mass index (BMI) than females (*p* < 0.001). The prevalence of overweight/obesity did not differ significantly between females and males (11.8% vs. 15.0%, respectively; *p* = 0.063).

As shown in Table 2, the majority of participants resided in rural areas (72.0%), whereas 18.2% lived in urban areas with 50,000–150,000 inhabitants. No significant sex differences were observed in place of residence (*p* = 0.653). Most participants reported engaging in regular physical activity, with 37.0% reporting physical activity several times per week and 42.8% once per week. Only 3.2% reported no physical activity. The frequency of physical activity did not differ significantly between females and males (*p* = 0.089).

Table 3 presents the daily consumption of individual beverage categories in the study population by sex. In the overall study population, water had the highest median intake (960 mL/day; IQR = 1320 mL). Males consumed significantly more water than females (1080 vs. 720 mL/day, *p* = 0.037). Significant sex differences were also observed for the consumption of reduced-fat milk (2%), skim milk, plant-based and flavored milk beverages, and coffee with milk and sugar, with females reporting higher intakes than males. Females also reported significantly higher consumption of 100% vegetable juice; however, the median intake remained 0 mL/day in both groups. Although energy drink consumption differed significantly between females and males (*p* = 0.034), the median intake was 0 mL/day in both groups, indicating a highly skewed distribution of energy drink intake. Although statistically significant sex differences were observed for some beverage categories with median intakes of 0 mL/day in both groups, the corresponding rank-biserial effect sizes were small, indicating that these distributional differences were of limited practical magnitude. No significant sex differences were observed for the remaining beverage categories. In the sensitivity analysis, beverage-specific intake was additionally expressed as a percentage of total daily beverage intake (Supplementary Table S2). Significant sex differences were observed for water, 100% vegetable juice, reduced-fat milk (2%), skim milk, plant-based and flavored milk beverages, energy drinks, SSBs, and coffee with milk and sugar. Females had a higher proportional contribution of 100% vegetable juice, reduced-fat milk, skim milk, plant-based and flavored milk beverages, energy drinks, SSBs, and coffee with milk and sugar, whereas males had a higher proportional contribution of water to total beverage intake. No significant sex differences were observed for the remaining beverage categories. An additional analysis of energy drink consumption frequency showed that 318 participants (19.3%) reported consuming energy drinks 2–3 times per week. This consumption pattern was significantly more common among females than males (22.6% vs. 17.2%, respectively; χ^2^ = 7.39, *p* = 0.007). No significant differences were observed according to age group (20.1% among participants aged 14–15 years vs. 19.1% among those aged 16–18 years; *p* = 0.650) or BMI category (20.8% among participants with overweight/obesity vs. 19.1% among those with normal weight/underweight; *p* = 0.551). In the multivariable logistic regression model including sex, age group, and BMI category, males had significantly lower odds of consuming energy drinks 2–3 times per week compared with females (OR = 0.706, 95% CI: 0.552–0.904; *p* = 0.006), whereas age group and BMI category were not significantly associated with this consumption pattern.

Table 4 presents the daily energy intake from individual beverage categories by sex. Differences were observed in energy intake from reduced-fat milk (2%), skim milk, plant-based and flavored milk beverages, and coffee with milk and sugar, with females exhibiting higher values than males. Significant differences were also observed for energy drinks and 100% vegetable juice; however, the median energy intake from these beverages was 0 kcal/day in both groups. For most beverage categories, the median daily energy intake was either 0 kcal/day or very low. Water was not included in this analysis because it does not provide energy. In the sensitivity analysis, energy intake from each beverage category was additionally expressed as a percentage of total daily energy intake from beverages (Supplementary Table S3). Significant sex differences were observed for 100% vegetable juice, skim milk, plant-based and flavored milk beverages, and coffee with milk and sugar (all *p* < 0.05). No significant sex differences were observed for the remaining beverage categories.

Table 5 presents the proportion of participants achieving ≥80% of the AI based on beverage intake according to the EFSA, IOM, and NIH criteria. The highest proportion of participants meeting the recommendations was observed according to the NIH criteria (65.8%), followed closely by EFSA (65.2%), whereas the lowest proportion was observed according to the IOM criteria (50.6%). Across all three recommendation systems, females were significantly more likely than males to meet the recommendations for total fluid intake (all *p* < 0.001). No significant differences were observed between age groups regardless of the recommendation system applied. BMI was significantly associated with meeting the recommendations only according to the IOM criteria, with participants classified as having normal weight or underweight more frequently meeting the recommendations than those with overweight/obesity (52.5% vs. 38.9%, *p* < 0.001). No significant differences between BMI categories were observed when applying the EFSA or NIH criteria.

Table 6 presents the results of the multivariable logistic regression analysis evaluating independent predictors of achieving ≥80% of the AI based on beverage intake according to the EFSA, NIH, and IOM criteria. Across all three models, sex was a significant independent predictor of meeting the defined adherence threshold based on beverage intake. Females had significantly higher odds than males according to the EFSA (OR = 1.84, 95% CI: 1.48–2.28), NIH (OR = 1.79, 95% CI: 1.44–2.23), and IOM (OR = 2.54, 95% CI: 2.07–3.12) criteria (all *p* < 0.001). Age group was significantly associated with the outcome only in the NIH model, with participants aged 16–18 years having lower odds of meeting the threshold than those aged 14–15 years (OR = 0.70, 95% CI: 0.55–0.91, *p* = 0.006). BMI category was significantly associated with meeting the defined adherence threshold only according to the IOM criteria, with participants with overweight/obesity having lower odds of meeting the threshold than those with normal weight/underweight (OR = 0.59, 95% CI: 0.44–0.80, *p* < 0.001). Physical activity showed a significant overall association with the outcome in the IOM model in the omnibus test (*p* = 0.041), whereas no significant overall associations were observed for the EFSA (*p* = 0.059) or NIH (*p* = 0.055) criteria. However, none of the individual physical activity categories differed significantly from the reference category of no physical activity. Model diagnostics indicated no evidence of multicollinearity, with variance inflation factors ranging from 1.01 to 1.03 across all three models. All models were statistically significant overall (*p* < 0.001). McFadden’s pseudo-R^2^ values were 0.0199 for the EFSA model, 0.0201 for the NIH model, and 0.0447 for the IOM model. The corresponding areas under the ROC curve (AUC) were 0.592, 0.595, and 0.637, respectively, indicating limited discriminatory performance.

## 4. Discussion

The present study is the first to apply the preliminarily validated Polish version of the BEVQ-PL questionnaire for a comprehensive assessment of beverage consumption among Polish adolescents from the Małopolska region [19]. Due to the lack of previous studies using this instrument in this population, the possibilities for direct comparison of the findings are limited. At the same time, the use of the BEVQ-PL enabled a simultaneous assessment of beverage consumption patterns, including the volume of beverages consumed, energy intake from beverages, and estimated adherence to the three reference systems based on beverage intake (EFSA, IOM, and NIH).

A key finding of the present study is that estimates of adherence based on beverage intake in epidemiological studies depend not only on the actual drinking behaviors of the study population but also on the reference system applied. The EFSA [14], IOM [15], and NIH [16] recommendations were included to provide complementary reference frameworks for the assessment of estimated adherence based on beverage intake. Among these, the Polish national recommendations [16] provide the most directly applicable reference for the present population, as they were developed specifically for the Polish population, while the EFSA and IOM recommendations enable comparison with European and international studies. The use of different reference systems may result in different estimates of adherence because of differences in the underlying reference values and assessment criteria [27,28]. Therefore, rather than identifying a universally superior reference system, the present analysis allows the findings to be interpreted primarily in the context of the Polish national recommendations while also facilitating comparison with studies using other commonly applied reference systems [2,18,29].

### 4.1. Water Intake

Water was the most frequently consumed beverage in the study population; however, a substantial proportion of participants did not meet the defined adherence threshold based on beverage intake. Boys consumed significantly more water than girls, which may partly reflect biological differences in fluid requirements. During adolescence, boys generally have greater body mass, higher fat-free mass, greater total body water content, and higher energy requirements, all of which are associated with increased water requirements and greater fluid losses [14,30]. Behavioral factors may also play an important role, including differences in physical activity levels, the frequency of beverage consumption during exercise, and preferences for specific beverage categories [1]. Nevertheless, despite their higher absolute water intake, boys were less likely than girls to meet the defined adherence threshold based on beverage intake. This observation indicates that greater water consumption does not necessarily translate into a higher probability of meeting the defined adherence threshold, as the reference values differ by sex and are generally higher for males. Therefore, water intake should be interpreted in the context of both the biological determinants of fluid requirements and sex-specific reference values rather than solely on the basis of the absolute volume of fluids consumed. Importantly, the sensitivity analysis based on the proportional contribution of individual beverage categories to total beverage intake showed that several sex differences persisted after accounting for differences in overall beverage volume. This finding suggests that the observed variation reflects not only total beverage intake but also sex-specific beverage consumption patterns. The higher proportion of females achieving the defined beverage-based adherence threshold may partly reflect the lower sex-specific AI values established for females. Therefore, this finding should not be interpreted as indicating better physiological hydration status among females. The present study assessed self-reported beverage intake and estimated adherence based on beverage intake, while objective biomarkers of hydration status were not measured.

Previous studies have shown that beverage choices during adolescence are influenced by physiological factors, sex, dietary preferences, family and school environments, and health awareness [1,10,31]. However, these factors were not assessed in the present study and therefore cannot be used to explain the observed sex differences.

The present findings are consistent with observations from other countries indicating that water remains the primary source of fluid intake among children and adolescents, although adherence to recommendations for total fluid intake remains insufficient. This is supported by the recent systematic review by Papaoikonomou et al. [1], which included studies published between 2004 and 2024 and demonstrated that only a limited number of populations achieved the recommended levels of fluid intake according to the EFSA or IOM criteria. These findings suggest that difficulties in meeting fluid intake recommendations represent a widespread public health challenge among children and adolescents. Similar observations were reported in the HELENA-CSS study involving adolescents from eight European countries, where water was the most frequently consumed beverage despite the substantial contribution of energy-containing beverages to total fluid intake [32].

The consistency of these findings across different populations suggests that inadequate adherence to fluid intake recommendations is a multifactorial phenomenon. In addition to biological water requirements, behavioral and environmental factors—including established beverage preferences, limited access to drinking water throughout the day, insufficient awareness of the importance of adequate hydration, and the influence of family and school environments—appear to play an important role. These factors may explain why inadequate adherence to fluid intake recommendations is consistently observed across many countries despite cultural and dietary differences [1,33].

Compared with previous Polish studies, the present findings appear to be more favorable. In the PLACE-19 study, more than 70% of Polish adolescents did not meet the recommendations for total fluid intake, whereas in the present study more than half of the participants met the recommendations regardless of the reference system applied. These differences may be attributed to the use of different beverage assessment tools, differences in the characteristics of the study populations, the period during which the studies were conducted, and the criteria used to evaluate adherence to fluid intake recommendations. This highlights that comparisons of epidemiological studies on hydration should consider not only differences between populations but also methodological aspects, as these may substantially influence the estimated prevalence of adherence to fluid intake recommendations [18]. Similar findings were reported by Kostecka et al. [34], who demonstrated that the majority of children aged 11–13 years did not meet the recommendations for fluid intake, confirming that inadequate hydration remains an important public health concern among Polish children and adolescents.

### 4.2. Energy Intake from Beverages

The present findings indicate that the assessment of beverage quality should not be limited solely to the volume of beverages consumed but should also take into account the energy provided by individual beverage categories. Unlike water, many beverages represent an additional source of energy, which is primarily derived from simple sugars. This is of considerable importance from the perspective of preventing diet-related diseases, as energy consumed in liquid form may elicit a weaker satiety response than energy obtained from solid foods, thereby promoting a positive energy balance and increasing the risk of overweight, obesity, type 2 diabetes, and cardiovascular diseases [35,36]. This mechanism is associated with the different physiological response of the gastrointestinal tract to liquid energy intake, including reduced stimulation of gastric mechanoreceptors and weaker activation of the gut–brain axis responsible for appetite regulation. Consequently, energy consumed in beverages is compensated for to a lesser extent during subsequent meals [37,38].

The present findings are partly consistent with those of the HELENA-CSS study, in which water was the primary source of fluid intake, whereas SSB contributed the largest proportion of beverage-derived energy [32]. In the present population of Polish adolescents, 100% fruit juice, whole milk (3.2% fat), and reduced-fat milk (2%) also contributed to beverage-derived energy, whereas sugar-sweetened beverages were not the predominant source. To further characterize these patterns, the contribution of individual beverage categories to total beverage-derived energy was calculated and is presented in Supplementary Table S3. Discrepancies between the present findings and those reported in the HELENA-CSS study may reflect variation in beverage assessment methods, study population characteristics, and the periods in which the studies were conducted. However, because total dietary energy intake from foods was not assessed in the present study, the proportion of total daily energy intake contributed by beverages could not be determined. Similar trends have also been observed in national studies. The PLACE-19 study demonstrated that despite water being the predominant beverage consumed, a substantial proportion of dietary energy still originated from SSBs [18]. SSBs and energy drinks remain regular components of the diet of a considerable proportion of Polish children and adolescents, highlighting the need for continued educational initiatives aimed at reducing the consumption of energy-containing beverages [39,40,41,42,43]. The present findings indicate that despite the observed changes in beverage consumption patterns, energy intake from beverages remains an important public health concern that requires further educational and preventive interventions [44,45,46].

### 4.3. Adherence to the EFSA, IOM, and NIH Recommendations

The simultaneous application of the EFSA, IOM, and NIH reference systems allowed the findings to be interpreted from complementary national and international perspectives. For Polish adolescents, the Polish national recommendations provide the most directly applicable reference framework, whereas the EFSA and IOM criteria facilitate comparisons with European and international studies. Importantly, differences between these reference systems extend beyond their numerical age- and sex-specific AI values. Water requirements cannot be defined with sufficient precision to establish an Estimated Average Requirement [EAR]; therefore, the recommended intakes are expressed as AIs and are derived from available data on habitual water intake, hydration status, and water balance in healthy populations. Differences in the populations and evidence considered, as well as in the methodological approaches adopted by the respective institutions, may consequently result in different reference values. Thus, differences in adherence observed across the three systems should be interpreted in the context of the underlying reference frameworks rather than solely as a consequence of different numerical thresholds [14,15,16].

The present findings are consistent with those reported in the recent systematic review by Papaoikonomou et al., which demonstrated that the majority of children and adolescents do not achieve the recommended fluid intake regardless of the reference criteria applied. The authors also emphasized that the lack of a harmonized reference system complicates comparisons between epidemiological studies conducted in different countries and may lead to different conclusions regarding the prevalence of inadequate fluid intake [1]. Similar observations were reported by Guelinckx et al. [10], who found that approximately 60% of children and adolescents did not meet the EFSA recommendations for fluid intake, with males and older participants being more likely to fail to achieve the recommended intake. These observations are consistent with the findings of the present study, which demonstrated that boys were less likely than girls to meet the defined adherence threshold based on beverage intake, regardless of the reference system applied.

The present findings indicate that estimates of adherence based on beverage intake should be interpreted in the context of the reference system used. Differences in reference criteria may limit the comparability of adherence estimates across studies and lead to different conclusions regarding the proportion of adolescents classified as meeting the defined adherence threshold. Importantly, the comparison of the three reference systems provides information beyond differences in the estimated prevalence of adherence. The multivariable analyses showed that the identification of factors associated with adherence was not entirely consistent across the reference systems. While sex was consistently associated with adherence, the association with BMI status was observed only when the IOM criteria were applied. Thus, the choice of reference system may influence not only the estimated prevalence of adequate fluid intake but also the identification of population subgroups considered at greater risk of inadequate intake. This finding has methodological implications for the interpretation and comparability of epidemiological studies using different fluid intake reference frameworks. Importantly, physical activity level was included simultaneously with BMI category in the multivariable logistic regression model. Therefore, the association between BMI category and adherence to the IOM recommendations was observed after adjustment for physical activity, suggesting that it was not solely attributable to differences in physical activity levels.

One possible explanation is that the higher AI values specified in the IOM [15] recommendations compared with those proposed by the EFSA [14] and the Polish Nutrition Standards [16] resulted in a more stringent classification of adherence. Consequently, differences in beverage consumption behaviors may have become more apparent when the IOM reference values were applied. More specifically, the higher IOM-derived threshold may have amplified differences in adherence between BMI categories by increasing the proportion of adolescents with lower beverage intake who were classified as not meeting the recommendations. This threshold effect may explain why overweight/obesity was significantly associated with lower odds of meeting the IOM recommendations, whereas the corresponding associations observed for the EFSA and NIH criteria did not reach statistical significance.

The absence of a similar association under the EFSA and NIH criteria suggests that the observed association between BMI and adherence to fluid intake recommendations may depend on the reference system applied rather than solely on actual differences in fluid consumption. Consequently, differences between anthropometric groups in the likelihood of meeting the intake criterion may be more pronounced when the IOM criteria are used than when the EFSA or NIH recommendations are applied [14,27,28].

Physical activity was also considered in the multivariable models because of its potential influence on fluid intake and hydration-related behaviors. A significant overall association between physical activity and adherence was observed only for the IOM criteria (*p* = 0.041), whereas the corresponding associations for the EFSA and NIH criteria did not reach statistical significance. However, none of the individual physical activity categories differed significantly from the reference group of adolescents reporting no physical activity. These findings suggest that the association between physical activity and adherence to fluid intake recommendations was limited and depended on the reference system applied. Female sex was also independently associated with adherence to total fluid intake recommendations in the logistic regression analysis. The estimated OR indicated that girls were approximately 1.8 to 2.5 times more likely than boys to achieve the recommended intake values, depending on the reference system applied, even after adjustment for the other variables included in the model. This association may reflect both the lower AI values established for girls and sex-specific beverage consumption patterns observed during adolescence [18]. Thus, despite consuming greater absolute volumes of water, boys may be less likely to meet the recommendations because the reference values for total fluid intake are substantially higher for males.

### 4.4. Health Implications of the Observed Beverage Consumption Patterns

The analysis of beverage consumption patterns indicates that water was the primary source of fluid intake in the study population, whereas energy-containing beverages, including 100% fruit juice, whole milk (3.2% fat), reduced-fat milk (2%), and sweetened tea, contributed to beverage-derived energy intake. These findings highlight the importance of considering both the volume and the types of beverages consumed when characterizing consumption patterns among adolescents. The additional analysis of energy drink consumption frequency showed that nearly one in five adolescents reported consuming these beverages 2–3 times per week. This consumption pattern was significantly more common among females than males, whereas no significant differences were observed according to age group or BMI status.

These findings have important public health implications. Energy consumed in liquid form may elicit a weaker satiety response than energy derived from solid foods and may be only partially compensated for during subsequent meals, thereby potentially promoting a chronic positive energy balance and increasing the risk of overweight, obesity, type 2 diabetes, non-alcoholic fatty liver disease, and cardiovascular diseases [35,47].

The observed beverage consumption pattern is consistent with findings from studies conducted in other populations, indicating that despite water being the predominant beverage consumed, energy-containing beverages remain an important component of the diets of children and adolescents [1,32]. The present findings may help identify beverage consumption patterns and population groups that could benefit from targeted nutrition and hydration education. Future studies conducted in nationally representative populations are warranted to confirm these observations and to support the development of evidence-based public health strategies. This suggests that preventive interventions should address the overall pattern of beverage consumption, including products that are often perceived as healthier options, such as 100% fruit juice and whole milk (3.2% fat), which may also contribute to beverage-derived energy intake. Such an approach is consistent with current international recommendations on nutrition for children and adolescents, which emphasize the importance of overall beverage consumption patterns for maintaining good health [48,49].

### 4.5. Strengths and Limitations

The present study has several important strengths. The relatively large sample size substantially exceeded the minimum required sample size, thereby increasing the precision of the estimates and the statistical power of the analyses. Another strength is the simultaneous evaluation of adherence according to three widely used reference systems (EFSA, IOM, and NIH), which enables a more comprehensive interpretation of the findings and facilitates comparisons with previous studies. In addition, anthropometric measurements were obtained using standardized procedures performed by trained research staff.

The study also has several limitations. Participants were recruited using a convenience sampling approach from secondary schools located in a single region of Poland; therefore, selection bias cannot be excluded, and the generalizability of the findings to the national adolescent population is limited. Seasonality was not explicitly accounted for in the statistical analyses; therefore, potential seasonal variation in beverage consumption cannot be excluded. In addition, participants were clustered within 15 schools, whereas the statistical analyses were conducted at the individual level without explicit adjustment for school-level clustering. Potential within-school correlation may therefore have affected the precision of the estimated associations, particularly the standard errors and confidence intervals in the logistic regression models. The multivariable results should consequently be interpreted as individual-level associations within the present sample. Future studies should consider multilevel or cluster-adjusted analytical approaches to account explicitly for the hierarchical structure of school-based data. Another limitation is that physical activity was assessed using self-reported data rather than objective measures, which may have introduced reporting bias. Beverage consumption was assessed using a self-administered questionnaire and may therefore be subject to recall bias and reporting bias. In particular, social desirability bias cannot be excluded, and its potential influence on sex-specific reporting patterns should be considered when interpreting the observed differences. Furthermore, the BEVQ-PL evaluates beverage consumption only and does not account for water obtained from foods; consequently, adherence was estimated using the assumption that beverages contribute approximately 80% of total water intake. Importantly, this proportion represents a population-level estimate and was not measured individually in the present study. Because the relative contribution of beverages and foods to total water intake may vary between individuals, applying a fixed ≥80% AI threshold may have resulted in some misclassification of participants as meeting or not meeting the recommendations. Accordingly, the reported adherence estimates should be interpreted as approximations of meeting beverage-based fluid intake targets rather than direct measures of compliance with total-water AI recommendations. Hydration status was not verified using objective biomarkers, such as urine osmolality or urine specific gravity. Therefore, the present study assesses self-reported beverage intake and estimated adherence based on beverage intake rather than physiological hydration status. Future studies combining the BEVQ-PL with objective hydration biomarkers could provide a more comprehensive assessment of the relationship between habitual beverage intake and hydration status. Furthermore, the BEVQ-PL assesses energy intake from beverages but does not provide information on total daily energy intake from the entire diet. Therefore, the proportion of total dietary energy contributed by individual beverage categories could not be calculated. Finally, the cross-sectional design precludes causal inference regarding the observed associations.

### 4.6. Challenges and Future Research Directions

The present findings provide an important contribution to understanding beverage consumption patterns and estimated adherence based on beverage intake among adolescents from the Małopolska region and may inform future nationwide studies. At the same time, the results highlight the need for further research in this area. Future studies should consider complementing self-reported beverage intake with objective biomarkers of hydration status, such as urine osmolality or urine specific gravity. This would allow verification of the agreement between reported fluid intake and objectively measured hydration status. In addition, future research should account for factors influencing fluid requirements, including physical activity level and environmental conditions, while also assessing water intake from foods, which would enable a more comprehensive interpretation of beverage-based adherence. The present study yielded different descriptive estimates of adherence according to the reference system applied. Therefore, studies simultaneously evaluating adherence according to the recommendations of the EFSA, IOM, and national nutrition guidelines would be particularly valuable. Such an approach would improve the comparability of studies conducted in different populations and facilitate the interpretation of epidemiological data on beverage intake and estimated adherence among children and adolescents.

Prospective studies are also warranted to evaluate the long-term effects of beverage consumption patterns during adolescence on nutritional status, metabolic health, and the risk of chronic diseases later in life. Studies employing standardized beverage assessment tools, such as the BEVQ-PL, would be particularly valuable, as they would enable the monitoring of changes in beverage consumption patterns over time and facilitate reliable comparisons between populations. The findings of such studies could contribute to the refinement of fluid intake recommendations and support the development of more effective public health strategies aimed at promoting appropriate beverage consumption among children and adolescents.

## 5. Conclusions

This study represents the first application of the validated BEVQ-PL questionnaire in a population of Polish adolescents, providing novel data on beverage consumption patterns and adherence to total fluid intake recommendations among adolescents from the Małopolska region according to three independent reference systems: EFSA, IOM, and NIH. The estimated proportions of adolescents meeting the defined beverage-based adherence threshold were 65.2% according to EFSA, 50.6% according to IOM, and 65.8% according to the Polish Nutrition Standards. The lower adherence observed according to the IOM criteria compared with the EFSA and NIH criteria illustrates how the choice of reference system may affect the estimated prevalence of adequate fluid intake. These findings highlight the importance of reporting the reference system applied when interpreting epidemiological findings and comparing results across populations.

Sex was the strongest factor associated with adherence to fluid intake recommendations, with girls being more likely than boys to meet the recommendations across all three reference systems. In addition, older age was associated with lower adherence only according to the NIH criteria, whereas overweight/obesity was associated with lower adherence only according to the IOM criteria.

The present findings may help identify adolescents who could benefit from targeted nutrition and hydration education programs, particularly those less likely to achieve the defined beverage-based adherence threshold. Furthermore, the observed differences in estimated adherence according to the reference system applied highlight the importance of clearly specifying the criteria used when assessing beverage-based adherence and when comparing findings across studies. The present study does not establish the relative validity or superiority of any of the reference systems; further research is needed to evaluate their comparative validity.

## Figures and Tables

**Table 1:** Comparison of AI recommendations for total water intake according to EFSA, IOM, and NIH guidelines.

Guideline	Age (Years)	Girls/Females (mL/Day)	Boys/Males (mL/Day)
EFSA [14]	14–18	2000	2500
IOM [15]	14–18	2300	3300
NIH (Poland) [16]	13–15	1950	2350
	16–18	2000	2500

**Table 2:** Sociodemographic and lifestyle characteristics of the study population by sex.

Variable	Overall (*n* = 1644)	Females (*n* = 660)	Males (*n* = 984)	*p*-Value
Age (years)	16.4 ± 1.1	16.4 ± 1.1	16.3 ± 1.1	0.003
BMI (kg/m^2^)	21.6 ± 3.4	21.0 ± 3.4	22.0 ± 3.3	<0.001
**BMI category**				
Underweight/normal weight (<85th percentile)	1418 (86.3%)	582 (88.2%)	836 (85.0%)	0.063
Overweight/obesity (≥85th percentile)	226 (13.7%)	78 (11.8%)	148 (15.0%)	
**Place of residence**				
Rural area (village)	1184 (72.0%)	474 (71.8%)	710 (72.2%)	0.653
Urban area (<50,000 inhabitants)	147 (8.9%)	54 (8.2%)	93 (9.5%)	
Urban area (50,000–150,000 inhabitants)	299 (18.2%)	125 (18.9%)	174 (17.7%)	
Urban area (150,000–500,000 inhabitants)	14 (0.9%)	7 (1.1%)	7 (0.7%)	
**Physical activity**				
No physical activity	53 (3.2%)	24 (3.6%)	29 (2.9%)	0.089
Occasionally (≤1 time/month)	278 (16.9%)	129 (19.5%)	149 (15.1%)	
At least once per week	704 (42.8%)	275 (41.7%)	429 (43.6%)	
Several times per week	609 (37.0%)	232 (35.2%)	377 (38.3%)	

Notes: Continuous variables are presented as mean ± standard deviation (SD) and categorical variables as *n* (%)**.** Differences between groups were assessed using the Mann–Whitney U test for continuous variables and Pearson’s chi-square test for categorical variables. Statistical significance was set at *p* < 0.05.

**Table 3:** Daily consumption of individual beverage categories in the study population by sex (mL/day).

Beverage Category	Overall Median (IQR)	Females Median (IQR)	Males Median (IQR)	*p*-Value
Water	960 (1320)	720 (1320)	1080 (1320)	0.037
100% Fruit Juice	43.2 (216)	50.4 (173)	33.6 (216)	0.223
Sweetened Fruit Drinks, Lemonades	33.6 (120)	33.6 (120)	16.8 (120)	0.196
100% Vegetable Juice	0.0 (0.0)	0.0 (16.8)	0.0 (0.0)	<0.001
Whole Milk (3.2% fat)	33.6 (130)	33.6 (130)	33.6 (130)	0.789
Reduced-Fat Milk (2%)	16.8 (120)	33.6 (130)	0.0 (120)	0.016
Skim Milk, Plant-Based, Flavored Milk Beverages	0.0 (0)	0.0 (33.6)	0.0 (0)	<0.001
Sugar-Sweetened Beverages (SSBs, e.g., soda, cola)	33.6 (130)	43.2 (130)	33.6 (130)	0.077
Energy Drinks	0.0 (33.6)	0.0 (43.2)	0.0 (33.6)	0.034
Diet Beverages with Sweeteners (e.g., zero-calorie beverages)	0.0 (86.4)	0.0 (86.4)	0.0 (86.4)	0.092
Sweetened Tea	86.4 (256)	86.4 (256)	86.4 (277)	0.940
Black Coffee, Tea	85.2 (256)	85.2 (256)	86.4 (256)	0.711
Coffee with Milk and Sugar	0.0 (130)	16.8 (170)	0.0 (130)	0.002
Smoothies, Protein Drinks	0.0 (86.4)	0.0 (94.8)	0.0 (85.2)	0.057

Notes: Data are presented as medians (IQRs). Differences between females and males were assessed using the Mann–Whitney U test. Statistical significance was set at *p* < 0.05.

**Table 4:** Comparison of daily energy intake (kcal/day) from individual beverage categories between females and males.

Beverage Category	Females Median (IQR), kcal/Day	Males Median (IQR), kcal/Day	*p*-Value
100% Fruit Juice	22.68 (77.80)	15.12 (97.20)	0.223
Sweetened Fruit Drinks, Lemonades	8.40 (30.00)	4.20 (30.00)	0.196
100% Vegetable Juice	0.00 (6.72)	0.00 (0.00)	<0.001
Whole Milk (3.2% fat)	21.50 (82.90)	21.50 (82.90)	0.789
Reduced-Fat Milk (2%)	16.80 (64.80)	0.00 (60.00)	0.016
Skim Milk, Plant-Based, Flavored Milk Beverages	0.00 (15.10)	0.00 (0.00)	<0.001
Sugar-Sweetened Beverages (SSBs, e.g., soda, cola)	18.10 (54.40)	14.10 (54.40)	0.077
Energy Drinks	0.00 (19.40)	0.00 (15.10)	0.034
Diet Beverages with Sweeteners (e.g., zero-calorie beverages)	0.00 (0.86)	0.00 (0.86)	0.092
Sweetened Tea	21.60 (63.90)	21.60 (69.20)	0.940
Black Coffee, Tea	1.70 (5.11)	1.73 (5.11)	0.711
Coffee with Milk and Sugar	6.72 (68.20)	0.00 (51.80)	0.002
Smoothies, Protein Drinks	0.00 (94.80)	0.00 (85.20)	0.057

Notes: Data are presented as medians and interquartile ranges (IQRs). Differences between females and males were assessed using the Mann–Whitney U test. Statistical significance was set at *p* < 0.05. Given the exploratory nature of the beverage-specific comparisons, *p*-values were not adjusted for multiple testing. Accordingly, statistically significant findings from these analyses should be interpreted as exploratory.

**Table 5:** Proportion of participants with beverage intake corresponding to ≥80% of the AI according to EFSA, IOM, and NIH criteria.

Variable	Category	EFSA *n* (%)	*p*-Value	IOM *n* (%)	*p*-Value	NIH *n* (%)	*p*-Value
Overall	Meets the recommendation	1072 (65.2)	-	832 (50.6)	-	1082 (65.8)	-
Sex	Females	483 (73.2)	<0.001	423 (64.1)	<0.001	483 (73.2)	<0.001
	Males	599 (60.9)		409 (41.6)		599 (60.9)	
Age	14–15 years	284 (67.1)	0.333	214 (50.6)	0.993	294 (69.5)	0.063
	16–18 years	788 (64.5)		618 (50.6)		788 (64.5)	
BMI	Underweight/normal weight	937 (66.1)	0.063	744 (52.5)	<0.001	945 (66.6)	0.076
	Overweight/obesity	135 (59.7)		88 (38.9)		137 (60.6)	

Notes: Data are presented as the number (*n*) and percentage (%) of participants achieving ≥80% of the AI based on beverage intake. Differences between groups were assessed using Pearson’s chi-square test. Statistical significance was set at *p* < 0.05. EFSA, IOM, and NIH recommendations were based on 80% of the AI for total water intake, assuming that approximately 20% of total water intake is provided by food.

**Table 6:** Logistic regression analysis of factors associated with achieving ≥80% of the AI from beverages according to the EFSA, NIH, and IOM criteria.

Predictor	EFSA OR (95% CI)	*p*-Value	NIH OR (95% CI)	*p*-Value	IOM OR (95% CI)	*p*-Value
Female vs. male	1.84 (1.48–2.28)	<0.001	1.79 (1.44–2.23)	<0.001	2.54 (2.07–3.12)	<0.001
16–18 years vs. 14–15 years	0.79 (0.62–1.01)	0.056	0.70 (0.55–0.90)	0.006	0.85 (0.67–1.08)	0.179
Overweight/obesity vs. underweight/normal weight	0.77 (0.58–1.04)	0.085	0.77 (0.57–1.03)	0.077	0.59 (0.44–0.80)	<0.001
Physical activity (overall)	-	0.059	-	0.055	-	0.041
Occasionally (≤1 time/month) vs. no physical activity	1.27 (0.69–2.33)	0.444	1.31 (0.72–2.41)	0.380	1.06 (0.58–1.95)	0.844
At least once per week vs. no physical activity	1.29 (0.72–2.29)	0.393	1.35 (0.76–2.40)	0.309	1.08 (0.61–1.92)	0.798
Several times per week vs. no physical activity	1.69 (0.94–3.02)	0.079	1.76 (0.98–3.15)	0.058	1.46 (0.81–2.61)	0.207

Notes: OR, odds ratio; 95% CI, 95% confidence interval. OR values > 1 indicate higher odds of achieving ≥80% of the AI based on beverage intake compared with the reference category, whereas OR values < 1 indicate lower odds. The multivariable logistic regression models included sex, age group, BMI category, and physical activity simultaneously. Reference categories were male, 14–15 years, normal weight/underweight, and no physical activity. For physical activity, overall *p*-values were obtained using likelihood-ratio omnibus tests. Statistical significance was set at *p* < 0.05.

## Data Availability

The data presented in this study are available upon request from the corresponding author due to privacy or ethical restrictions.

## Supplementary Materials

The following supporting information can be downloaded at https://www.mdpi.com/article/10.3390/nu18172784/s1, Table S1: Estimated energy density coefficients (kcal/mL) assigned to beverage categories for BEVQ-PL scoring based on Polish market data; Table S2: Contribution of individual beverage categories to total daily beverage intake by sex; Table S3: Proportional contribution of individual beverage categories to total daily energy intake from beverages according to sex.

## Abbreviations

The following abbreviations are used in this manuscript:

AI	Adequate Intake
BEVQ-PL	Polish Beverage Intake Questionnaire
BMI	Body Mass Index
CI	Confidence Interval
EFSA	European Food Safety Authority
IOM	Institute of Medicine
IQR	Interquartile Range
kcal	Kilocalorie
mL	Milliliter
NIH	National Institute of Public Health—National Institute of Hygiene (Poland)
OR	Odds Ratio
SD	Standard Deviation
SSBs	Sugar-Sweetened Beverages
STROBE	Strengthening the Reporting of Observational Studies in Epidemiology
TBW	Total Body Water
TWI	Total Water Intake
