# Supplementary material for: Beverage Consumption Patterns, Beverage-Derived Energy Intake, and Adherence to Fluid Intake Recommendations Among Polish Adolescents: A Cross-Sectional Study Using the BEVQ-PL Questionnaire
Source: Nutrients. 2026 Aug 26;18(17):2784. doi: 10.3390/nu18172784 (PMC13567327; doi:10.3390/nu18172784)
Supplement: Supplementary file 1 [file nutrients-18-02784-s001.zip › nutrients-4506641-supplementary.pdf]

## Supplementary Tables

**Supplementary Table S1: Estimated energy density coefficients (kcal/mL) assigned to beverage categories for BEVQ-PL scoring based on Polish market data.**

| Beverage Category | Energy density kcal/mL |
|---|---|
| Water | 0.00 |
| 100% Fruit Juice | 0.45 |
| Fruit Drinks / Lemonades | 0.40 |
| 100% Vegetable Juice | 0.25 |
| Whole Milk (3.2% fat) | 0.64 |
| Reduced-Fat Milk (2%) | 0.50 |
| Skim Milk / Plant-Based / Flavored Milk | 0.45 |
| Diet Beverages with Sweeteners (e.g., zero-calorie beverages) | 0.42 |
| Energy Drinks | 0.45 |
| Diet Beverages with Sweeteners e.g., zero-calorie beverages | 0.01 |
| Sweetened Tea | 0.25 |
| Black Coffee or Tea | 0.02 |
| Coffee with Milk and/or Sugar | 0.40 |
| Smoothies / Protein Drinks | 0.60 |

**Notes:** Values were assigned to beverage categories based on representative products available on the Polish market and used to estimate beverage-derived energy intake in the BEVQ-PL scoring algorithm.

**Supplementary Table S2: Contribution of individual beverage categories to total daily beverage intake by sex.**

| Beverage category | Females Median (IQR), % | Males Median (IQR), % | *p*-value |
|---|---|---|---|
| Water | 38.99 (41.2) | 44.18 (43.7) | 0.015 |
| 100% Fruit juice | 2.35 (7.01) | 1.94 (7.33) | 0.214 |
| Fruit Drinks / Lemonades | 1.12 (4.28) | 0.85 (4.14) | 0.125 |
| 100% Vegetable juice | 0.00 (1.13) | 0.00 (0.00) | <0.001 |
| Whole milk (3.2% fat) | 1.27 (5.75) | 1.30 (6.01) | 0.674 |
| Reduced-fat milk (2%) | 1.27 (5.39) | 0.00 (4.85) | 0.019 |
| Skim Milk / Plant-Based / Flavored Milk | 0.00 (1.33) | 0.00 (0.00) | <0.001 |

| | | | |
|---|---|---|---|
| Sugar-Sweetened Beverages (e.g., soda, cola) | 1.81 (6.14) | 1.38 (5.08) | 0.034 |
| Energy drinks | 0.00 (1.85) | 0.00 (0.99) | 0.022 |
| Diet Beverages with Sweeteners (e.g., zero-calorie beverages) | 0.00 (3.38) | 0.00 (3.20) | 0.076 |
| Sweetened tea | 3.45 (11.5) | 3.75 (12.2) | 0.997 |
| Black Coffee or Tea | 2.69 (11.6) | 3.45 (11.1) | 0.604 |
| Coffee with Milk and/or Sugar | 0.67 (5.77) | 0.00 (4.92) | 0.001 |
| Smoothies / Protein Drinks | 0.00 (3.58) | 0.00 (2.85) | 0.052 |

**Notes:** Data are presented as median (interquartile range, IQR). Values represent the percentage contribution of each beverage category to total daily beverage intake. Differences between females and males were assessed using the Mann–Whitney U test. Statistical significance was set at $p < 0.05$.

**Supplementary Table S3: Proportional contribution of individual beverage categories to total daily energy intake from beverages according to sex.**

| Beverage category | Females, Median (IQR), % | Males, Median (IQR), % | p-value |
|---|---|---|---|
| 100% Fruit Juice | 4.93 (17.90) | 5.33 (19.00) | 0.737 |
| Fruit Drinks / Lemonades | 1.37 (5.03) | 1.08 (5.42) | 0.560 |
| 100% Vegetable Juice | 0.00 (1.91) | 0.00 (0.00) | <0.001 |
| Whole Milk (3.2% fat) | 4.25 (18.20) | 5.01 (21.90) | 0.148 |
| Reduced-Fat Milk (2%) | 2.93 (13.30) | 1.21 (12.70) | 0.073 |
| Skim Milk / Plant-Based / Flavored Milk | 0.00 (2.99) | 0.00 (0.00) | <0.001 |
| Sugar-Sweetened Beverages (e.g., soda, cola) | 4.01 (11.90) | 3.09 (11.40) | 0.112 |
| Energy Drinks | 0.00 (3.92) | 0.00 (2.29) | 0.057 |
| Diet Beverages with Sweeteners (e.g., zero-calorie beverages) | 0.00 (0.16) | 0.00 (0.17) | 0.277 |
| Sweetened Tea | 3.83 (13.50) | 4.40 (17.00) | 0.568 |
| Black Coffee or Tea | 0.22 (1.26) | 0.32 (1.47) | 0.591 |
| Coffee with Milk and/or Sugar | 1.55 (10.20) | 0.00 (9.13) | 0.007 |
| Smoothies / Protein Drinks | 0.00 (8.26) | 0.00 (7.39) | 0.149 |

**Notes:** Data are presented as median and interquartile range (IQR). Percentages represent the contribution of energy from each beverage category to total daily energy intake from beverages. Differences between females and males were assessed using the Mann–Whitney U test. Statistical significance was set at $p < 0.05$.
